# Dual EGFR and BRAF blockade overcomes resistance to vemurafenib in BRAF mutated thyroid carcinoma cells

**DOI:** 10.1186/s12935-017-0457-z

**Published:** 2017-10-04

**Authors:** Tiziana Notarangelo, Lorenza Sisinni, Valentina Condelli, Matteo Landriscina

**Affiliations:** 1 Laboratory of Pre-Clinical and Translational Research, IRCCS, Referral Cancer Center of Basilicata, Via Padre Pio, 1, Rionero in Vulture, 85028 Italy; 2 Medical Oncology Unit, Department of Medical and Surgical Sciences, University of Foggia, Viale Pinto, 1, Foggia, 71100 Italy

**Keywords:** BRAF, Thyroid carcinoma, EGFR, Vemurafenib, Gefitinib

## Abstract

**Background:**

BRAF inhibitors are effective anticancer agents in BRAF-mutated melanomas. By contrast, evidences about sensitivity of thyroid carcinomas to BRAF inhibition are conflicting and it has been proposed that BRAF V600E thyroid carcinoma cells are less sensitive to BRAF inhibitors due to activation of parallel signaling pathways. This study evaluated the hypothesis that feedback activation of EGFR signaling counteracts the cytostatic activity of vemurafenib (PLX4032) in BRAF V600E thyroid carcinoma cells.

**Methods:**

Cell proliferation, cell cycle distribution, induction of apoptosis and EGFR and AKT signaling were evaluated in thyroid carcinoma cell lines bearing the BRAF V600E mutation in response to PLX4032.

**Results:**

A partial and transient cytostatic response to PLX4032 was observed in thyroid carcinoma cell lines bearing the BRAF V600E mutation, with lack of full inhibition of ERK pathway. Interestingly, the exposure of thyroid carcinoma cells to PLX4032 resulted in a rapid feedback activation of EGFR signaling with parallel activation of AKT phosphorylation. Consistently, the dual inhibition of EGFR and BRAF, through combination therapy with PLX4032 and gefitinib, resulted in prevention of EGFR phosphorylation and sustained inhibition of ERK and AKT signaling and cell proliferation. Of note, the combined treatment with gefitinib and vemurafenib or the exposure of EGFR-silenced thyroid carcinoma cells to vemurafenib induced synthetic lethality compared to single agents.

**Conclusions:**

These data suggest that the dual EGFR and BRAF blockade represents a strategy to by-pass resistance to BRAF inhibitors in thyroid carcinoma cells.

**Electronic supplementary material:**

The online version of this article (doi:10.1186/s12935-017-0457-z) contains supplementary material, which is available to authorized users.

## Background

Cancers bearing BRAF mutations represent approximately 8% of all human malignancies, these mutations occurring more frequently in melanomas (40–70%), and thyroid (36–53%), colorectal (5–22%) and low grade serous ovarian (~ 30%) carcinomas [1, 2]. In such a context, approximately 90% of BRAF mutations result in the substitution of glutamic acid for valine at position 600 (BRAF V600E) [1]. Indeed, the oncogenic activation of BRAF leads to constitutive activation of downstream signaling through MAPK pathway [3] and favors the development of biologically and clinically aggressive thyroid and colorectal malignancies, frequently resistant to conventional anticancer therapies [4, 5].

Inhibition of the BRAF V600E oncoprotein by small-molecule drugs, such as vemurafenib (PLX4032) or PLX4720, results in marked antitumor activity in human melanoma cells carrying the BRAF V600E mutation [6]. However, other human malignancies (i.e., thyroid and colorectal carcinomas) are less sensitive to BRAF inhibitors (BRAFi), regardless BRAF mutational status [7, 8]. Among several mechanisms responsible for resistance, it has been suggested that most tumors who initially respond to BRAFi eventually develop acquired resistance through activation of alternative pathways leading to reactivation of cell proliferation [7, 8]. Indeed, the exposure of colorectal cancer cells to BRAFi results in a feedback activation of EGFR and lack of sensitivity to vemurafenib [9]. Prerequisite for development of this mechanism of drug resistance is the upregulation of EGFR, since melanoma cells devoid of EGFR expression are sensitive to vemurafenib, lacking this feedback activation, and the ectopic expression of EGFR induces resistance to PLX4032 in melanoma cells [9]. Thus, inhibition of EGFR signaling by monoclonal antibodies (i.e., Cetuximab) or tyrosine kinase inhibitors (TKi; i.e., gefitinib or erlotinib) is synergistic with BRAF inhibition in colon carcinoma cells [9]. Consistently with a role of HER receptor family in resistance to BRAFi, Montero-Conde et al. reported that BRAF-mutated thyroid carcinoma (TC) cells exposed to PLX4032 are characterized by transient inhibition of ERK phosphorylation with rebound activation of HER3 signaling. Indeed, the pan-HER TKi lapatinib prevents ERK rebound and sensitizes BRAF-mutant thyroid cancer cells to RAF or MAPK kinase inhibitors [10].

Based on this premise, this study evaluated the hypothesis that the exposure of BRAF-mutated TC cells to vemurafenib results in EGFR feedback activation and that dual EGFR and BRAF blockade is superior to single agents. This issue is extremely relevant in a clinical perspective, since human TCs are characterized by high expression of EGFR and poor responsiveness to EGFR inhibitors [11]. In addition, 25–50% of thyroid cancers are BRAF mutated and constitutive activation of BRAF signaling leads to aggressive malignancies, lacking typical traits of thyroid differentiation [2, 12] and, thus, poorly responsive to radioiodine therapy [13].

## Methods

### Cell cultures, siRNAs and chemicals

Papillary BCPAP, poorly differentiated WRO, anaplastic BHT101 and FRO TC cell lines were purchased from DSMZ (Braunschweig, Germany). BHT101, FRO and BCPAP cell lines are characterized by the BRAF V600E mutation, being WRO cells wild type for BRAF gene [14]. Cell line authentication was verified before starting this study by STR profiling, according to ATCC product description, and by BRAF mutational status. All cell lines were cultured in DMEM containing 10% (v/v) fetal bovine serum (FBS), 2 mM glutamine, and 100 U/mL penicillin and streptomycin. BHT101 cells were cultured in the same medium supplemented with 20% (v/v) FBS.

Unless otherwise specified, reagents were purchased from Sigma-Aldrich (Milan, Italy). BRAF inhibitor PLX4032 (vemurafenib) was purchased from Selleck Chemicals (Huston, USA). Gefitinib was kindly provided by AstraZeneca, pertuzumab was kindly provided by Roche (Basel, Switzerland). Drugs were dissolved in dimethylsulfoxide (DMSO) and the same DMSO volume was added to untreated control.

SiRNA of EGFR was purchased from Qiagen (siRNA Cat. No. GS1956). For control experiments, cells were transfected with a similar amount of negative siRNA (Qiagen, Cat. No. SI03650318). For knock-down experiments, siRNAs were diluted to a final concentration of 40 nM and transiently transfected by using the HiPerFect Transfection Reagent (Qiagen), according to manufacturer’s protocol.

### Growth curves and MTT assay

Growth rates were assessed upon seeding of cells in six-well plates at the concentration of 4 × 10^4^ cells/well. Cell lines were incubated in the presence and the absence of specified drug concentrations, harvested after 24, 48 and 72 h and counted in a Burker chamber (three countings per sample). Incubation with drugs was carried out continuously, and drug containing fresh medium was changed at 48 h intervals.

Cell viability was evaluated using the dimethylthiazol diphenyltetrazolium bromide (MTT) (Sigma-Aldrich, Italy) dye assay as previously described [15]. Briefly, cells were seeded into 24-well plates (1 × 10^4^ cells/well) and treated as described in Figure Legends. After drug removal, cells were incubated in a drug-free medium for 48 h, and, subsequently, in presence of 600 μM MTT solution for additional 3 h at 37 °C to allow MTT metabolism into formazan crystals. The formazan crystals were finally solubilized by adding 200 µL of 0.04 N HCl in isopropanol to each microplate well. Adsorbance at 540 nm was measured using a Bio-Tek microplate reader (model EL-340; BioMetallics, Priceston, NJ). Wells containing only DMEM, FBS and MTT were used as controls. Each experiment was performed three times using four replicates for each drug concentration.

### Cell cycle analysis

Cells were incubated in a culture medium supplemented with 20 mmol/L 5-bromo-20-deoxyuridine (BrdUrd) for 20 min and harvested. Subsequent to incubation in a solution containing 3 N HCl for 30 min at room temperature to obtain DNA denaturation, cell pellets were further incubated in the presence of anti-BrdUrd FITC (Becton–Dickinson) for 1 h at room temperature in the dark. After washing with PBS, cells were further incubated with 6 mg/mL propidium iodate (PI) for 20 min and then evaluated using FACSCalibur™ (Becton–Dickinson) [16].

### Immunoblot analysis

Total cell lysates were obtained by the homogenization of cell pellets in a cold lysis buffer (20 mmol/L Tris, pH 7.5 containing 300 mmol/L sucrose, 60 mmol/L KCl, 15 mmol/L NaCl, 5% (v/v) glycerol, 2 mmol/L EDTA, 1% (v/v) Triton X-100, 1 mmol/L PMSF, 2 mg/mL aprotinin, 2 mg/mL leupetin, and 0.2% (w/v) deoxycholate) for 2 min at 4 °C and further sonication for 30 s on ice. Immunoblot analysis was performed as previously reported [17]. The following antibodies from Santa Cruz Biotechnology were used: mouse monoclonal anti-GAPDH (sc-47724), rabbit polyclonal anti-phosphoEGFR (Tyr1173, sc-12351). The following antibodies from Cell Signaling Technology were also used: mouse monoclonal anti-phospho44/42 MAPK (pErk1/2, #9106), rabbit polyclonal anti-phosphoAKT (Ser473, #9271), rabbit polyclonal anti-AKT (#9272), rabbit polyclonal anti-EGFR (#4267). Rabbit polyclonal anti-MAPK (Erk1/2, #ABS44) antibody was purchased from Millipore Merck.

### Apoptosis assay

Apoptosis was evaluated by citofluorimetric analysis of Annexin-V and 7-amino-actinomycin-D (7-AAD)-positive cells using the fluorescein isothiocyanate (FITC)-Annexin-V/7-AAD kit (Beckman Coulter, Milan, Italy). Stained cells were analyzed using the FACSCalibur™ (Becton–Dickinson). Positive staining for Annexin-V as well as double staining for Annexin-V and 7-AAD were interpreted as signs of early and late phases of apoptosis respectively [18].

### Statistical analysis

The paired Student’s *t* test was used to establish the statistical significance between different levels of growth rate, cell cycle distribution and apoptosis compared to the respective controls. Statistically significant values (P < 0.05) are reported in Figure Legends. All experiments were independently performed at least three times.

## Results

### Vemurafenib partially inhibits cell growth and ERK signaling in thyroid carcinoma cells

**Fig. 1:**
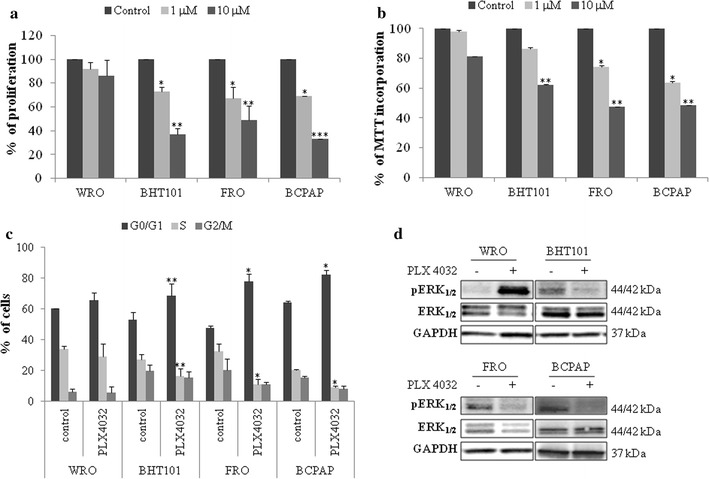
Vemurafenib partially inhibits cell proliferation, cell cycle progression and ERK signaling in BRAF V600E thyroid carcinoma cell lines. **a**, **b** Growth rate (**a**) and cell viability (**b**) in BRAF V600E BHT101, FRO and BCPAP thyroid carcinoma cell lines and BRAF-wild type WRO cells treated with 1 and 10 μM PLX4032. **c** Cell cycle distribution in BRAF V600E BHT101, FRO and BCPAP thyroid carcinoma cell lines and BRAF-wild type WRO cells exposed to 10 μM PLX4032 for 15 h. **a**–**c** Statistical significance respect to each untreated control: *p < 0.05; **p < 0.005; ***p < 0.0005. **d** ERK and phosphoERK immunoblot analysis in BRAF V600E BHT101, FRO and BCPAP thyroid carcinoma cell lines and BRAF-wild type WRO cells exposed to 10 μM PLX4032 for 1 h

To establish the sensitivity of thyroid cancer cell lines to BRAFi, growth rate (Fig. 1a), cell viability (Fig. 1b) and cell cycle distribution (Fig. 1c) were evaluated in response to PLX4032 in TC cell lines harboring the BRAF V600E mutation (i.e., BHT101, FRO and BCPAP cells) or BRAF-wild type WRO cells. Indeed, PLX4032 was ineffective in BRAF wild-type WRO cells (Fig. 1a, b) and induced a dose dependent inhibition of cell growth in BRAF V600E BHT101, FRO and BCPAP cell lines (Fig. 1a, b). Noteworthy, PLX4032 inhibition of BRAF-mutated TC cell lines proliferation was incomplete, reaching a maximum of 60% downregulation of cell growth (Fig. 1a, b). Consistently, the exposure of BRAF V600E BHT101, FRO and BCPAP cells to PLX4032 resulted in the accumulation of cells in G0-G1 phase with a parallel attenuation of S phase (Fig. 1c) and this correlated with inhibition of ERK phosphorylation (Fig. 1d). As expected, the exposure of BRAF-wild type WRO cells to PLX4032 was ineffective in delaying cell cycle progression (Fig. 1c) and induced a paradoxical activation of ERK phosphorylation (Fig. 1d).

### BRAF inhibition results in feedback activation of EGFR phosphorylation in BRAF V600E thyroid carcinoma cells

**Fig. 2:**
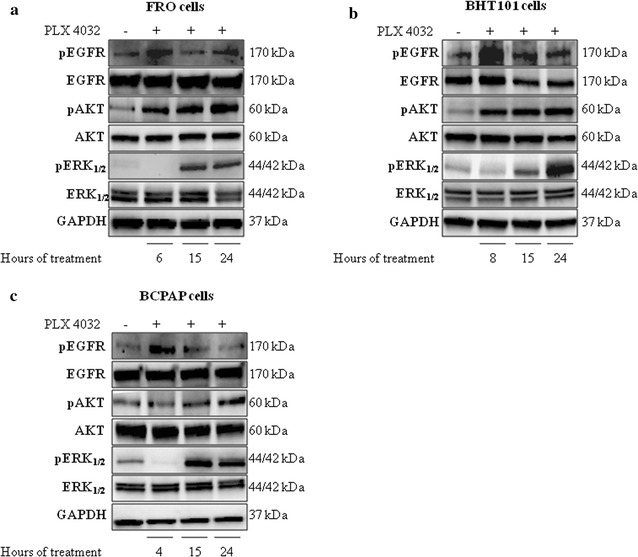
Vemurafenib induces a feedback activation of EGFR phosphorylation in BRAF V600E thyroid carcinoma cells. **a**–**c** EGFR, phosphoEGFR, AKT, phosphoAKT, ERK and phosphoERK immunoblot analysis in BRAF V600E FRO (**a**), BHT101 (**b**) and BCPAP (**c**) thyroid carcinoma cell lines exposed to 10 μM PLX4032 for 4–8, 15 and 24 h

Since these data suggest that BRAF V600E TC cells lines are not fully responsive to vemurafenib, the hypothesis that BRAF pharmacological inhibition results in feedback activation of EGFR signaling was further evaluated in BRAF-mutated BHT101, BCPAP and FRO cell lines. Thus, TC cells were exposed to 10 μM PLX4032 for short (4–8 h depending on the cell line) or longer (15 and 24 h) periods and evaluated for EGFR phosphorylation (Fig. 2). Interestingly, vemurafenib treatment induced a rapid feedback activation of EGFR phosphorylation between 4 and 8 h (Fig. 2a–c), this suggesting that EGFR signaling is induced by BRAF inhibition in BRAF-mutated TC cell lines. In addition, the kinetic of ERK signaling inhibition in response to PLX4032 showed a rapid and sustained reactivation of ERK signaling after 15–24 h of treatment (Fig. 2a–c). Consistently, vemurafenib induced the rebound activation of AKT phosphorylation, which occurred early in FRO and BHT101 cells (Fig. 2a, c) and at later time points in BCPAP cells (Fig. 2b). These data suggest that vemurafenib induces rebound activation of EGFR signaling in BRAFV600E TC cells.

### Dual EGFR and BRAF blockade induces inhibition of cell proliferation, suppression of ERK signaling and synthetic lethality

**Fig. 3:**
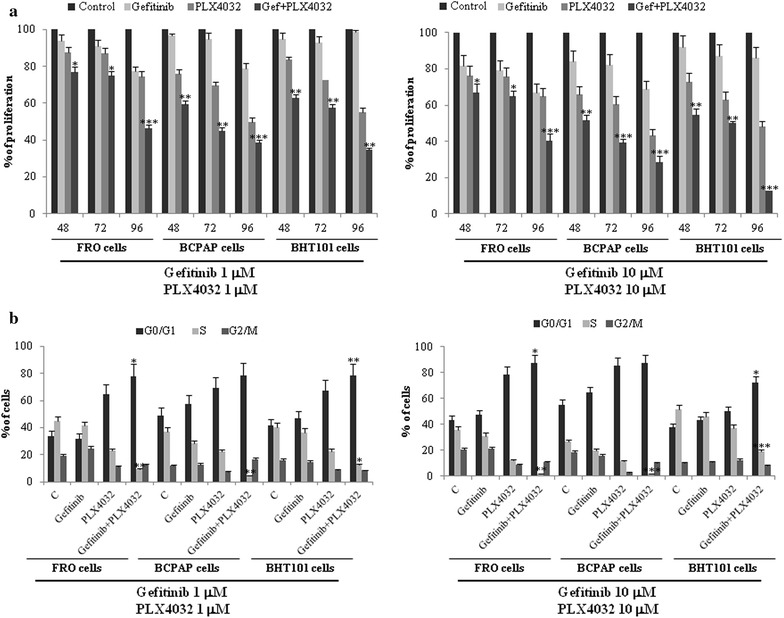
Dual EGFR and BRAF blockade inhibits cell proliferation and cell cycle progression in BRAF V600E thyroid carcinoma cells. **a**, **b** Cell growth (**a**) and cell cycle distribution (**b**) in BRAF V600E FRO, BCPAP and BHT101 thyroid carcinoma cell lines exposed to 1–10 μM PLX4032 or 1–10 μM gefitinib or the combination of both agents for indicated time points. Cell cycle distribution was evaluated after 24 h treatment. Statistical significance respect to vemurafenib single agent: *p < 0.05; **p < 0.005; ***p < 0.0005

**Fig. 4:**
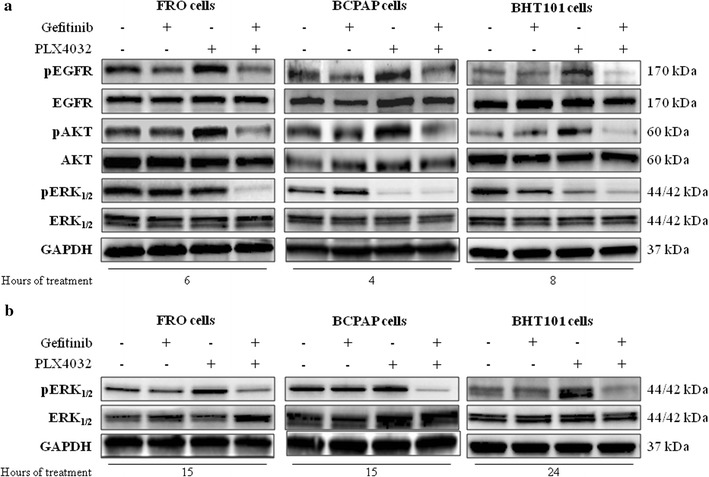
Dual EGFR and BRAF blockade prevents EGFR feedback activation and induces prolonged suppression of ERK signaling in BRAF V600E thyroid carcinoma cells. **a** AKT, phosphoAKT, EGFR, phosphoEGFR, ERK and phosphoERK immunoblot analysis in BRAF V600E FRO, BCPAP, and BHT101 thyroid carcinoma cell lines exposed to 10 μM PLX4032 or 10 μM gefitinib or the combination of both agents for 4 (BCPAP cells), 6 (FRO cells) or 8 (BHT101 cells) h. **b** ERK and phosphoERK immunoblot analysis in BRAF V600E FRO, BCPAP and BHT101 thyroid carcinoma cell lines exposed to 10 μM PLX4032 or 10 μM gefitinib or the combination of both agents for 15 h (24 h in BHT101 cells)

In further experiments, the hypothesis that dual blockade of EGFR and BRAF signaling results in potentiation of BRAFi single agent activity was further evaluated. Thus, the cytostatic activity of combined therapy with 1–10 μM PLX4032 and the EGFR inhibitor, gefitinib (1–10 μM) was evaluated in comparison with PLX4032 or gefitinib single agents in BRAF-mutated BHT101, FRO and BCPAP TC cell lines (Fig. 3). Indeed, the combined blockade of EGFR and BRAF resulted in a more significant inhibition of cell proliferation (Fig. 3a) and cell cycle progression, with increased accumulation of cells in G0–G1 phase and attenuation of S phase (Fig. 3b). Noteworthy, the combined inhibition of EGFR and BRAF prevented the feedback activation of EGFR phosphorylation (Fig. 4a) and the parallel activation of AKT phosphorylation (Fig. 4a) and induced a prolonged suppression of ERK signaling (Fig. 4b). Since the feedback activation of HER3 signaling is involved in acquired resistance to vemurafenib [10] and HER3 pathway is activated upon heterodimerization with HER2 receptor, this activation being blocked by pertuzumab [19], the inhibition of HER2/HER3 heterodimerization by pertuzumab was tested in combination with BRAFi and compared to the dual blockade of BRAF and EGFR in TC cells. Interestingly, the dual treatment with pertuzumab and vemurafenib resulted in a cytostatic activity comparable to the dual blockade of EGFR and BRAF (Additional file 1: Figure S1).

**Fig. 5:**
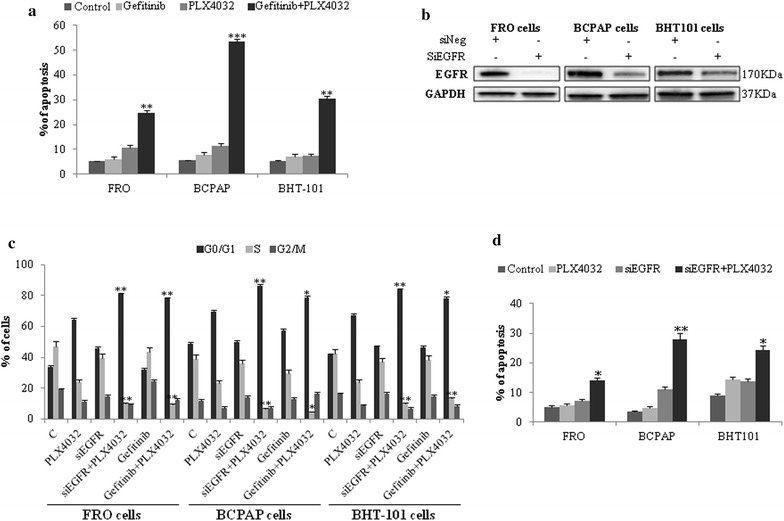
Dual EGFR and BRAF blockade induces synthetic lethality in BRAF V600E thyroid carcinoma cells. **a** Apoptotic cell death in BRAF V600E FRO, BCPAP and BHT101 thyroid carcinoma cell lines exposed to 10 μM PLX4032 or 10 μM gefitinib or the combination of both agents for 24 h (48 h in BHT101 cells). Statistical significance respect to vemurafenib single agent: *p < 0.01; **p < 0.001; ***p < 0.0001. **b** EGFR immunoblot analysis in FRO, BCPAP and BHT101 cells transfected with control (siNeg) or EGFR siRNA. **c**, **d** Cell cycle distribution (**c**) and apoptotic cell death (**d**) in BRAF V600E FRO, BCPAP and BHT101 thyroid carcinoma cell lines transfected with control (siNeg) or EGFR siRNA and subsequently exposed to 10 μM PLX4032 for 24 h. Statistical significance respect to vemurafenib single agent: *p < 0.01; **p < 0.001; ***p < 0.0001

Finally, the hypothesis that the combined blockade of EGFR and BRAF results in synthetic lethality was evaluated. Interestingly, while gefitinib and PLX4032 single agents exhibited no or minimal cytotoxic activity, the exposure of FRO, BCPAP and BHT101 TC cells to combination therapy with gefitinib and PLX4032 resulted in 10–40% induction of apoptotic cell death (Fig. 5a). The specificity of cytostatic and cytotoxic activity of combination therapy with gefitinib and vemurafenib was confirmed upon EGFR silencing and subsequent exposure to vemurafenib in FRO, BCPAP and BHT101 (Fig. 5b). Of note, vemurafenib induced a more significant cell cycle arrest (Fig. 5c) and higher levels of apoptosis (Fig. 5d) in a low EGFR background. These data support the concept that the dual blockade of EGFR and BRAF results in increased cytostatic activity and induction of synthetic lethality compared to BRAFi single agent.

## Discussion

Early responses involved in adaptive resistance to BRAFi have been described in human cancer cells [20]. Among several proposed mechanisms, the occurrence of genomic events, as secondary NRAS mutations and BRAF alternative splicing or other alterations, both upstream and downstream to BRAF, leading to reactivation of ERK pathway, provided the rational for combination treatment with BRAF inhibitors and other agents to circumvent or delay resistance [21]. In addition, the activation of alternative pathways are also responsible for resistance to BRAFi, leading mostly to selection of resistant clones that cause tumor regrowth and progressive disease [8]. This issue is extremely relevant in BRAF-mutated TCs that are characterized by loss of thyroid specific characters [12] and poor responsiveness to radioiodine therapy [13] and, thus, require new therapeutic options. In such a context, evidences about sensitivity of BRAF V600E TCs to vemurafenib single agent are conflicting with initial studies showing sustained responses [22, 23] and subsequent reports showing transient activity [24, 25].

This study was designed to evaluate the role of feedback activation of EGFR signaling in counteracting the cytostatic activity of vemurafenib in BRAF-mutated TC cell lines. Our data suggest that (1) BRAF V600E TC cell lines are transiently responsive to vemurafenib, (2) vemurafenib treatment elicits a feedback activation of EGFR pathway and (3) simultaneous blockade of BRAF and EGFR results in potentiation of vemurafenib single agent activity, prolonged suppression of ERK and AKT signaling and induction of synthetic lethality.

This evidence is consistent with previous studies, in colon carcinoma and melanoma cells, suggesting that EGFR expression dictates the activity of BRAF inhibitors. Indeed, melanoma cells with poor EGFR background are highly responsive to BRAF inhibitors, whereas colon carcinoma cells exhibit a rapid feedback activation of EGFR phosphorylation, being characterized by high EGFR expression [9]. Consistently, the upregulation of EGFR in melanoma cells results in loss of activity of BRAF inhibitors and dual inhibition of EGFR and BRAF re-establishes vemurafenib activity in colon carcinoma cells [9]. In such a context, TCs are characterized by high expression of EGFR and sustained activation of its downstream signaling [11], this representing a mechanism of escape from EGFR inhibitors [11]. Consistently, our data suggest that transient exposure of BRAF V600E TC cells to vemurafenib results in a rapid phosphorylation of EGFR and this correlates with reactivation of ERK and AKT signaling. Intriguingly, the simultaneous blockade of EGFR and BRAF results in a more significant and prolonged suppression of ERK and AKT signaling and induction of synthetic lethality compared to single agents. The specificity of this pharmacological activity is supported by data obtained with transient silencing of EGFR expression and subsequent treatment with vemurafenib, which results in arrest of cell cycle progression and synthetic lethality.

An issue that needs to be discussed is whether there is a relationship between EGFR rebound activation and AKT and ERK phosphorylation in response to vemurafenib. Indeed, our data show that AKT phosphorylation occurs at earlier time points compared to ERK re-activation, which is a later event. Interestingly, combined exposure of TC cells to gefitinib and vemurafenib results in sustained suppression of both AKT and ERK signaling in parallel with inhibition of EGFR phosphorylation, this suggesting that these events are likely to be part of a common adaptive response. While this issue deserves further investigation, much evidence supports the relevance of AKT activation in driving poor response to inhibitors of the RAF/RAS/MEK axis in TC cells lines. Indeed, AKT pathway is highly active in BRAF-mutated TC cell lines [26] and its phosphorylation is enhanced by inhibition of MEK/ERK signaling, regardless the BRAF mutational status [27]. In addition, the simultaneous suppression of MEK/ERK and PI3K/AKT pathways abrogates compensatory mechanisms of tumor survival and causes synergistic cytotoxicity in TC cell lines [27].

It is noteworthy that the combined blockade of HER2/HER3 heterodimerization and BRAF signaling is comparable to the combination of gefitinib and vemurafenib in terms of cytostatic activity. Indeed, Montero-Conde et al. reported that exposure of BRAF-mutated TC cells to PLX4032 results in rebound activation of HER3 signaling and inhibition of HER family receptor with lapatinib prevents ERK rebound activation and sensitizes BRAF-mutant TC cells to RAF or MAPK kinase inhibitors [10]. Thus, it is likely that feedback activation of multiple signaling pathways, most of them involving the HER family receptor, is responsible for resistance to BRAFi in TC cells.

Clinically relevant is the observation that combined inhibition of EGFR and BRAF signaling is more effective than vemurafenib or gefitinib single agents and results in induction of synthetic lethality. Indeed. BRAF-mutated TCs are aggressive malignancies frequently poor responsive to radioiodine therapy [13]. In such a perspective, BRAF/MEK/ERK signaling is emerging as a potential target is these malignancies. The BRAF inhibitor, dabrafenib and the MEK inhibitor, solumetinib were evaluated for their capacity to re-induce iodine uptake in iodine-refractory BRAF-mutated human TCs with potentially interesting results [28, 29]. In addition, the multi-targeted TK inhibitors sorafenib and lenvantinib obtained the approval by FDA as effective treatments in these malignancies [30]. In such a complex scenario, our data provide a rationale for evaluating dual EGFR and BRAF blockade as potential therapeutic option in BRAF-mutated radioiodine-refractory TCs. Notably, this strategy already provided interesting results in other human malignancies at either preclinical or clinical level. Indeed, pharmacological agents blocking EGFR signaling combined with BRAFi inhibited orthotopic glioma xenografts and increased apoptosis, with resultant significant extension of animal survival [31]. In addition, vemurafenib in combination with cetuximab and irinotecan showed valuable clinical activity and a reasonable toxicity profile in pretreated metastatic colorectal carcinomas [32].

## Conclusion

This study suggests that vemurafenib single agent activity is significantly impaired in BRAF V600E TC cells by feedback activation of EGFR signaling pathway and that dual inhibition of EGFR and BRAF may represent a strategy to potentiate BRAFi single agents.

## Additional file

**Additional file 1: Figure S1.** Cell cycle distribution in BRAF V600E FRO and BCPAP thyroid carcinoma cell lines exposed to 10 μM PLX4032, 10 μM gefitinib, 1 μM pertuzumab or the combination of PLX4032 with both agents for 24 h. Statistical significance respect to vemurafenib single agent: **p < 0.001.
