# Supplementary figures and images for: Dual EGFR and BRAF blockade overcomes resistance to vemurafenib in BRAF mutated thyroid carcinoma cells
Source: Cancer Cell Int. 2017 Oct 4;17:86. doi: 10.1186/s12935-017-0457-z (PMC5628448; doi:10.1186/s12935-017-0457-z)

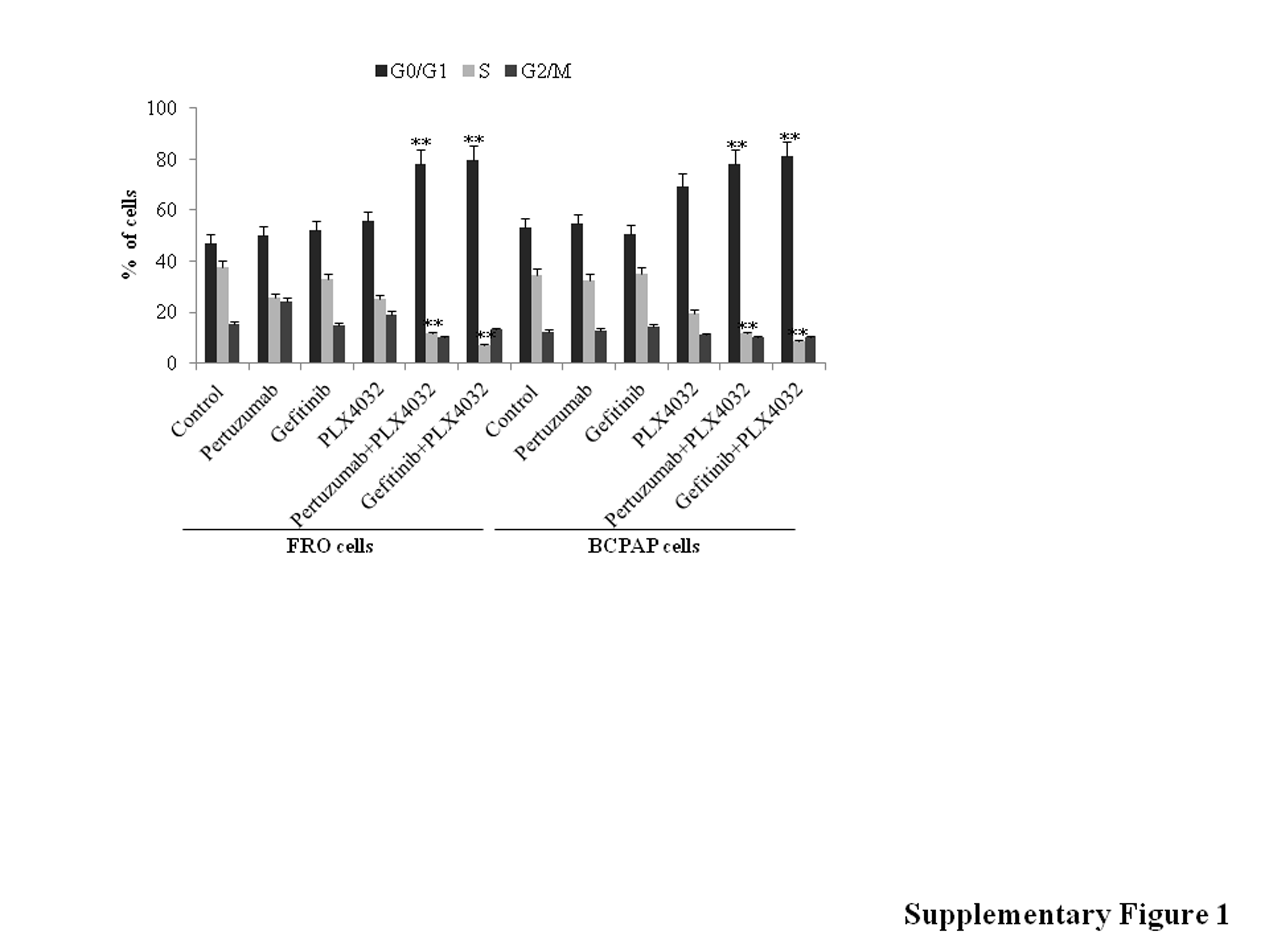

Supplement: Supplementary file 1 — Additional file 1: Figure S1. Cell cycle distribution in BRAF V600E FRO and BCPAP thyroid carcinoma cell lines exposed to 10 μM PLX4032, 10 μM gefitinib, 1 μM pertuzumab or the combination of PLX4032 with both agents for 24 h. Statistical significance respect to vemurafenib single agent: **p < 0.001. [file 12935_2017_457_MOESM1_ESM.tif]
